# Thermal plasticity is independent of environmental history in an intertidal seaweed

**DOI:** 10.1002/ece3.5796

**Published:** 2019-11-18

**Authors:** Sophie J. McCoy, Stephen Widdicombe

**Affiliations:** ^1^ Department of Biological Science Florida State University Tallahassee FL USA; ^2^ Plymouth Marine Laboratory Plymouth UK

**Keywords:** algae, environmental history, global change ecology, photosynthesis, plasticity, thermal stress

## Abstract

Organisms inhabiting the intertidal zone have been used to study natural ecophysiological responses and adaptations to thermal stress because these organisms are routinely exposed to high‐temperature conditions for hours at a time. While intertidal organisms may be inherently better at withstanding temperature stress due to regular exposure and acclimation, they could be more vulnerable to temperature stress, already living near the edge of their thermal limits. Strong gradients in thermal stress across the intertidal zone present an opportunity to test whether thermal tolerance is a plastic or canalized trait in intertidal organisms. Here, we studied the intertidal pool‐dwelling calcified alga, *Ellisolandia elongata*, under near‐future temperature regimes, and the dependence of its thermal acclimatization response on environmental history. Two timescales of environmental history were tested during this experiment. The intertidal pool of origin was representative of long‐term environmental history over the alga's life (including settlement and development), while the pool it was transplanted into accounted for recent environmental history (acclimation over many months). Unexpectedly, neither long‐term nor short‐term environmental history, nor ambient conditions, affected photosynthetic rates in *E. elongata*. Individuals were plastic in their photosynthetic response to laboratory temperature treatments (mean 13.2°C, 15.7°C, and 17.7°C). Further, replicate ramets from the same individual were not always consistent in their photosynthetic performance from one experimental time point to another or between treatments and exhibited no clear trend in variability over experimental time. High variability in climate change responses between individuals may indicate the potential for resilience to future conditions and, thus, may play a compensatory role at the population or species level over time.

## Graphical abstract

We studied the intertidal pool‐dwelling calcified alga, *Ellisolandia elongata*, under near‐future temperature regimes, and the dependence of its thermal acclimatization response on environmental history. Two timescales of environmental history were tested during this experiment: the pool of origin was representative of long‐term environmental history over the alga's life (including settlement and development), while the pool it was transplanted into accounted for recent environmental history (acclimation over many months). Unexpectedly, neither long‐term nor short‐term environmental history, nor ambient conditions, affected photosynthetic rates in *E. elongata* and individuals were plastic in their photosynthetic response to laboratory temperature treatments.

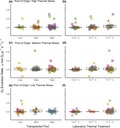

## 1 INTRODUCTION

Thermal tolerance lies at the core of many processes in ecology, from ecophysiological mechanisms to macroecological patterns (Bartsch, Vogt, Pehlke, & Hanelt, 2013; Helmuth, Broitman, et al., 2006; Helmuth, Mieszkowska, Moore, & Hawkins, 2006; Hutchins, 1947; Somero, 2005; Vernberg, 1962). Over the next century, ocean surface temperatures are anticipated to raise up to 0.3°C per decade (Alexander et al., 2018). Temperature changes throughout this range are likely to engender changes in community structure and functioning (Schindler, 1990), including shifts in population dynamics and species abundances in temperate marine communities (Hale, Calosi, McNeill, Mieszkowska, & Widdicombe, 2011; Queirós et al., 2015).

The intertidal zone, where marine organisms are exposed to terrestrial conditions sustained for hours at a time, has been used to study natural ecophysiological response and adaptations to thermal stress (Breeman, 1988; Davenport & Davenport, 2005; Egilsdottir, Olafsson, & Martin, 2015; Harley et al., 2006; Helmuth, Broitman, et al., 2006; Helmuth et al., 2002; Helmuth, Mieszkowska, et al., 2006). Intertidal organisms have also been proposed as better suited to withstand climate changes, due to their existence in environments that fluctuate temporally in temperature, irradiance, and other chemical factors (Egilsdottir, Noisette, Noël, Olafsson, & Martin, 2013; Harley et al., 2012; Raven, Giordano, Beardall, & Maberly, 2012). However, these organisms live close to their thermal tolerance limits (Bertness, Leonard, Levine, & Bruno, 1999; Davenport & Davenport, 2005; Doty, 1946; Fields, Graham, Rosenblatt, & Somero, 1993; Hofmann & Somero, 1995; Tomanek & Helmuth, 2002; Wethey, 1983) and instead may be more likely to reveal effects of climate extremes on marine organisms (Barry, Baxter, Sagarin, & Gilman, 1995; Bertness et al., 1999; Fields et al., 1993; Helmuth, Broitman, et al., 2006; Helmuth et al., 2002; Helmuth, Mieszkowska, et al., 2006; Lima, Ribeiro, Queiroz, Hawkins, & Santos, 2007; Sagarin, Barry, Gilman, & Baxter, 1999; Southward, Hawkins, & Burrows, 1995). Indeed, the strong zonation patterns exhibited by intertidal organisms suggests that they may be adapted only to the particular temperature excursions that they experience locally—during periods of low tide or isolation of tide pools from the surrounding seawater—and that are associated with a specific tidal height (Axelsson & Uusitalo, 1988; Davison & Pearson, 1996; Johnson, Gigon, Gulmon, & Mooney, 1974; Murru & Sandgren, 2004; Smith & Berry, 1986).

Phenotypic plasticity describes environmentally induced phenotypic variation (sensu Stearns, 1989). Changes in environmental conditions can affect phenotypic development (Price, Qvarnstrom, & Irwin, 2003; West‐Eberhard, 2003). Over longer timescales of sustained changes to the environment, genetic accommodation should result either in genetic assimilation, where environmentally induced phenotypes become genetically canalized even in the absence of the environmental stimulus (Pigliucci & Murrena, 2003), or in genetic compensation, where canalization does not occur and the phenotype remains sensitive to environmental cues (Grether, 2005). Where stress is short‐term, then reversible phenotypic plasticity will be selected for (Moran, 1992; Pigliucci, 2001; Scheiner, 1993). While reversible phenotypic plasticity may seem like the optimal solution to maximize fitness over the largest range of environmental conditions, its evolution may be constrained by genetics, energetics, timescale, or otherwise. In such cases, a nonplastic phenotype shifted toward tolerance of the environmental stressor is likely to evolve (Gabriel, 2005). Thus, in the case of intertidal species, we hypothesized that such developmental canalization would be likely to occur in populations that repeatedly experience predictable thermal stress on diurnal and seasonal scales, such as experienced by intertidal organisms. We further hypothesized that trait canalization may differ between individuals inhabiting intertidal pools at different tidal heights, which experience different extremes in thermal stress.

Calcified algae have shown variable responses to warming experiments (Cornwall, Diaz‐Pulido, & Comeau, 2019; Jokiel et al., 2008; Kuffner, Andersson, Jokiel, Rodgers, & Mackenzie, 2008; Martin & Gattuso, 2009; Nannini, Marchi, Lombardi, & Ragazzola, 2015). This potentially points to high plasticity in this algal group that thrives across a variety of marine environments, including in highly fluctuating coastal and intertidal environments (McCoy & Kamenos, 2015; Schaum & Collins, 2014). Much evidence for intertidal stress or reduced physiological performance is derived more from aerial exposure during low tide than from temperature or nutrient excursions in tidal pools (Ji & Tanaka, 2002; Martone, Alyono, & Stites, 2010; McCoy, Pfister, Olack, & Colman, 2016; Mueller, Fischer, Bolch, & Wright, 2015). Within the articulated coralline algae, comparison between a subtidal species and an intertidal species found submerged in tide pools revealed that only the intertidal pool‐dwelling species was able to recover from both thermal and desiccation stress (Guenther & Martone, 2014). Additionally, the tide pool‐dwelling alga photosynthetically outperformed the subtidal alga under both high‐ and low‐tide conditions, which simulated warming water in pools during low tide (Guenther & Martone, 2014). Intertidal algae are generally more productive than subtidal algae during favorable environmental conditions, exhibiting greater photosynthetic activity despite having similar concentrations of photosynthetic pigments (Guenther & Martone, 2014), continuing to calcify at night and when aerially exposed (Egilsdottir et al., 2015; McCoy et al., 2016), and having greater activity of carbon concentrating mechanisms (Murru & Sandgren, 2004; Raven & Osmond, 1992; Stepien, 2015). These traits point to adaptations of intertidal macroalgae to maximize productivity under ideal conditions and during periods of stress.

Macroalgal photosynthesis is temperature‐dependent, as temperature directly influences diffusion rates and other metabolic rates, including synthesis of photosynthetic pigments (Flukes, Wright, & Johnson, 2015; Hurd, Harrison, Bischof, & Lobban, 2014). In addition to short‐term temperature dependence of photosynthetic traits, there is also evidence that photosynthetic processes may acclimate to temperature (Zou & Gao, 2014). In this study, we thus tested the effects of long‐term environmental history over the organism's life (including settlement and development) and recent environmental history (acclimation over many months) on the thermal tolerance of the perennial intertidal alga, *Ellisolandia elongata*, as determined by its photosynthetic rate. Photosynthetic rate was chosen because of its temperature dependence in macroalgae and because it is a proxy for primary productivity and growth (Littler & Arnold, 1980), which in turn serve as fitness proxies in macroalgae (Dethier & Steneck, 2001; Pfister, 1992).

The articulated coralline *E. elongate* abounds in intertidal pools across the United Kingdom (Brodie, Walker, Williamson, & Irvine, 2013) and supports many associated organisms by providing chemical and physical habitat, acting as a refuge from the temperature and moisture mosaics of the intertidal (Jones, Lawton, & Schachak, 1994; Nelson, 2009). Therefore, the sensitivity of *E. elongate* to future temperature regimes will likely influence the success of associated rock pool fauna. More broadly, the response of *E. elongata* yields insights into processes of plasticity in response to varying environmental conditions. This study aimed to test the effects of timescales of environmental history on the thermal tolerance of *E. elongata* to study processes of trait canalization in an ecologically important taxon.

## 2 MATERIALS AND METHODS

*Ellisolandia elongate* (J. Ellis & Solander) K.R. Hind & G.W. Saunders (Brodie et al., 2013; Hind & Saunders, 2013) is a geniculate coralline red alga found along the southwest coasts of England and Ireland (Brodie et al., 2013, as *Corallina elongata*). Geniculate coralline algae are long‐lived perennial species with typically high growth and colonization rates, forming algal “turfs” consisting of upright, branched, geniculate fronds stemming from a basal thallus that adheres to rocky substrate (reviewed in McCoy & Kamenos, 2015). As in other coralline algae, size of the basal thallus is positively related to age (Dethier & Steneck, 2001). Collected specimens included the basal thallus still attached to rocky substrate, with healthy, mature fronds emerging from across the basal crust (approximately 4‐cm x 4‐cm basal crust area). Thus, collected specimens were all estimated to be at least 1 year old.

### 2.1 Field transplants

**Figure 1:**
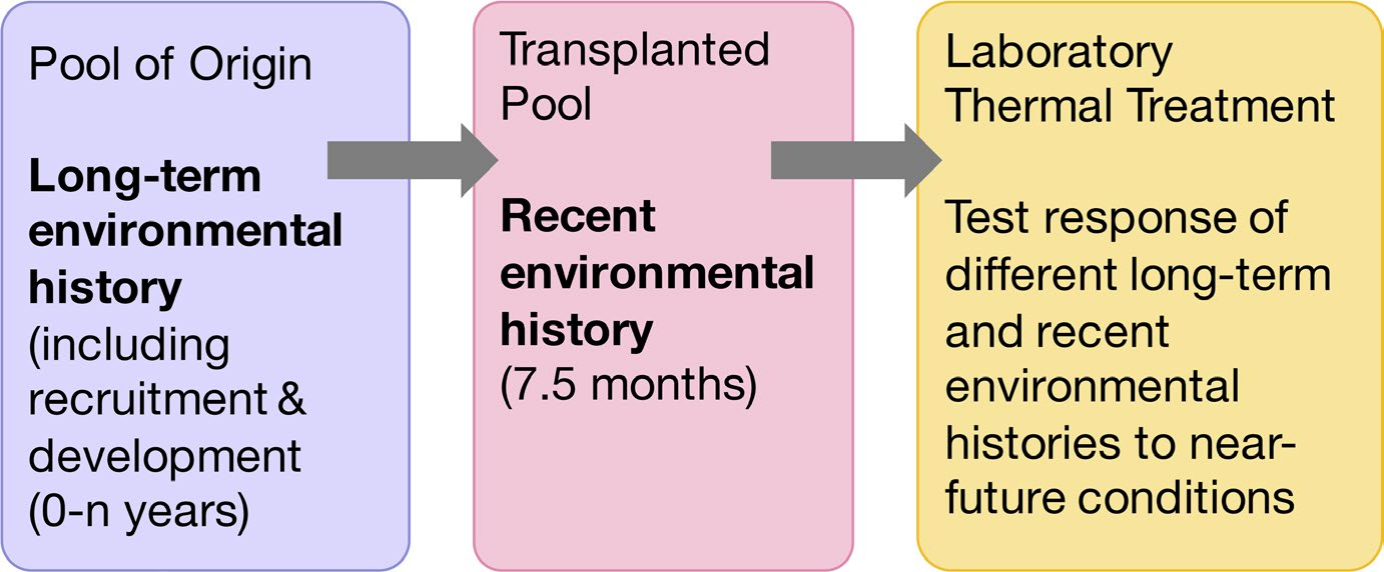
Flowchart of the experimental design. *Ellisolandia elongata* was sampled from its pool of origin, representative of long‐term environmental history. Three pools in each low, medium, and high thermal stress categories were chosen across the intertidal zone. Samples were reciprocally transplanted among these pools, representative of recent environmental history for 7.5 months. Transplanted algae were removed from the field and tested in the laboratory under thermal conditions representative of current, +50 years and +100 years mean sea surface temperatures to determine effects of long‐term and recent environmental history on responses to near‐future thermal conditions

Transplants along a strong intertidal gradient were done to set up variation in long‐term (pool of origin) and recent (transplanted pool) environmental history (Figure 1). Twenty‐seven turf samples of *E. elongata* were collected from nine tidal pools at Cape Cornwall, Penzance, England (50°07′44.8″N, 5°42′16.4″W) using hammer and chisel on October 28, 2015. Pools were chosen to be representative of a gradient in thermal stress, with smaller pools located high in the tidal range (upshore) representative of the highest thermal stress, and large, low‐shore pools experiencing the lowest thermal stress. Three tidal pools were chosen within each category of low, medium, and high thermal stress, and three turf samples of *E. elongata* were collected from each pool for transplantation.

After sample collection, tidal pools were partially drained to allow installation of HOBO temperature and irradiance loggers (Onset Corp.) below the waterline of each pool at low tide. Collected samples were kept in outdoor buckets overnight and reciprocally transplanted using marine epoxy (Z‐SPAR, A‐788 Splash Zone) on 29 October 2015 after emptied pools had refilled naturally over the tidal cycle. Transplants from each pool were dispersed between low‐, medium‐, and high‐stress pools, including samples transplanted back into their original “home” pool of collection (i.e., out of three samples collected from a low‐stress pool, one was returned to its original pool, one was transplanted to a medium‐stress pool, and one was placed in a high‐stress pool).

At the end of the field portion of the study (221 days), photosynthetic rates of *E. elongata* native to each pool (not manipulated in experimental transplants) were measured in ambient summer sunlight unobstructed by clouds in the morning (08:00 GMT, mean irradiance 1,186 ± 781 Lux, mean pool temperature 12.8 ± 0.1°C) and in the afternoon (15:00 GMT, mean irradiance 103,402 ± 30,896 Lux, mean pool temperature 21.0 ± 0.5°C) on 6 June 2016.

Evolution of O_2_ gas in seawater was measured over 12 min using a four‐channel FireStingO_2_ oxygen meter fitted with air‐tight 4‐ml vials containing fiber‐optic sensors (PyroScience). During each incubation, one vial was incubated with seawater from the tidal pool without an algal sample as a seawater blank. Each of the remaining three vials contained one frond that was plucked at its base from a nontransplanted individual within the tidal pool and was filled with ambient pool water. Algal fronds used in each incubation were collected and air‐dried at the laboratory for 1 week prior to weighing, allowing O_2_ evolution to be normalized to dried sample mass.

### 2.2 Laboratory experiments

On 6 June 2016, transplanted *E. elongata* samples were removed from the field after 221 days and acclimated to laboratory conditions at 13.2°C in 1‐m^3^ recirculating seawater tanks at Plymouth Marine Laboratory overnight. The following day, algae were separated into replicate ramets by chiseling epoxy disks and were placed across temperature treatment tanks. Replicate ramets from the same transplanted individual were labeled using colored thread.

Experimental tanks were held at control (mean 13.2°C), medium (mean 15.7°C) and high (mean 17.7°C) temperature treatments using electric heaters. Temperature treatments were chosen to match ambient seawater temperatures at the time of sample collection in June (~13°C) and aimed to recreate predicted seawater temperatures in 50 (~15°C) and 100 years (~17°C), respectively (Alexander et al., 2018). Water temperature was measured twice daily and adjusted manually if temperature deviated by >0.2°C. Approximately 10% of the water mass was exchanged each week with freshly collected seawater from the L4 Station of the Western Channel Observatory (50°15.0′N, 4°13.0′W).

Once per week, evolution of O_2_ gas in seawater was measured over 12 min using a four‐channel FireStingO_2_ oxygen meter fitted with air‐tight 4‐mL vials containing fiber‐optic sensors (PyroScience) for each sample in the laboratory at photosynthetically active radiation (PAR) averaging 27.2 ± 0.5 photosynthetic photon flux density, mimicking PAR levels in tide pools at high tide in summer in this region (Kolzenburg et al., 2019). For each tank, one seawater blank was measured using a vial incubated with seawater from the tank without an algal sample and used as a correction for all measurements from that tank. To measure algal photosynthesis, one frond was plucked at its base from each transplant and placed in a vial filled with ambient treatment water. Each incubated algal frond was rinsed with distilled water and air‐dried for 1 week prior to weighing and used to normalize O_2_ evolution to dried sample mass.

### 2.3 Statistical analyses

Differences between temperatures in pools from different thermal stress categories were tested by ANOVA over the entire transplant period from October 2015 to June 2016 (R statistical program; R Core Team, 2017). Temperature differences during February 2016, the coldest temperature exposure during the study period, and during May 2016, the warmest and most recent temperature history of the study period, were also tested with ANOVA by pool size.

ANOVA was used to test for differences in time of day field‐measured photosynthetic rates, looking for differences only between pools that were sampled both in the morning and in the afternoon. All pools were sampled in the morning and were grouped by thermal stress category to test for differences in morning photosynthetic rates between thermal stress groups.

A nested ANOVA design (linear mixed model) was used to test for changes in photosynthetic rate over time within a laboratory temperature treatment, which was tested on each temperature group separately, with individual identity as a fixed effect and tank nested within temperature treatment (“lmer” fitted with REML, R package “lme4” v. 1.1‐21; Bates et al., 2019). Next, we tested for differences between temperature treatments within each timepoint, separating our dataset by week for these analyses, using a nested ANOVA design with treatment temperature and individual identity as fixed effects and with the random effect of tank nested within each treatment. Random effects are tested using REML‐likelihood ratio tests with Type III Satterthwaite error (“ranova” function, R package “lmerTest” v. 3.1–0; Kuznetsova, Brockhoff, & Christensen, 2019).

We highlight the most extreme comparison in our analysis, using an ANOVA design to ask whether high‐stress acclimatized ramets differ from low‐stress acclimatized ramets under stressful laboratory conditions. This consists of ramets originally from pools of either high or low thermal stress, transplanted to their pool of origin (i.e., identical long‐ and short‐term thermal history), and subsequently placed in the low and high laboratory temperature treatments.

Standard deviation between replicates of each individual (dispersed as replicates in each temperature treatment) was calculated at each time point. Within‐individual standard deviation was regressed over experimental time to determine whether within‐individual variability decreased with time in the temperature treatments.

## 3 RESULTS

### 3.1 Pools as a thermal stress gradient

**Figure 2:**
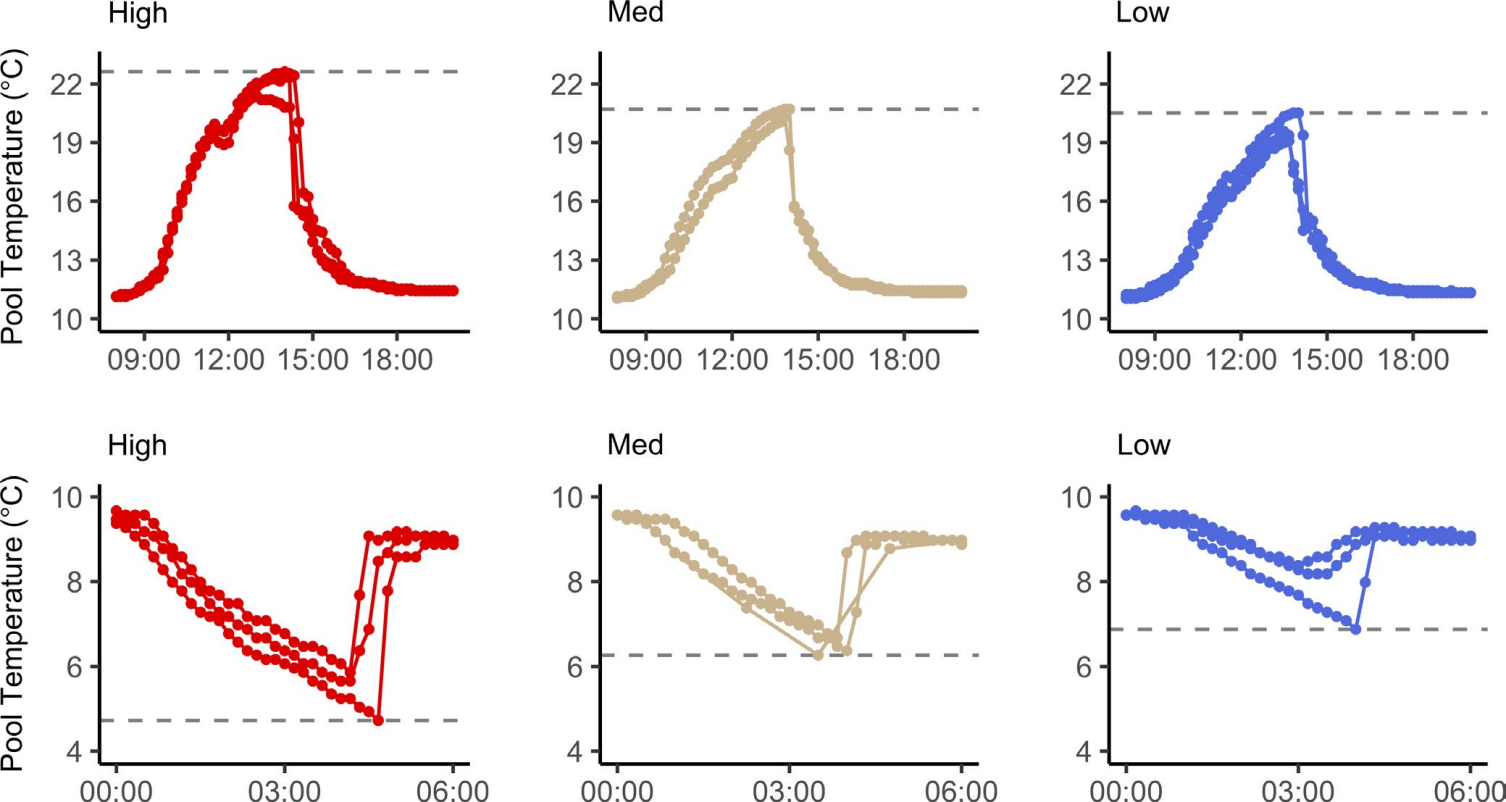
Temperature within tide pools during low‐tide events associated with thermal stress, showing high‐temperature events in the top row (8 May 2016) and low‐temperature events in the bottom row (29 February 2016). Each panel is labeled categorically by pool for low (blue), medium (tan), and high (red) thermal stress, and each line represents a different pool replicate. Horizontal lines in each panel denote maximum (top row) or minimum (bottom row) temperatures observed in each tide pool

Over the transplant period (221 days, 28 October 2015–6 June 2016), minimum pool temperature was colder (*F*_2,6_ = 13.04, *p* = .007) and maximum pool temperature was hotter (*F*_2,6_ = 10.05, *p* = .012) in smaller, higher pools, with temperature extremes down to 4.7°C and up to 23.9°C occurring during low tide (Figure 2, Table 1). This pattern was maintained when testing February 2016 pool temperatures during the coldest period of the year (minimum temperature, *F*_2,6_ = 12.11, *p* = .008, maximum temperature, *F*_2,6_ = 2.16, *p* = .196) and in May 2016 during the hottest period (minimum temperature, *F*_2,5_ = 20.21, *p* = .004, maximum temperature, *F*_2,5_ = 11.1, *p* = .015, Table 2). Thus, pools provided a template for different thermal environments.

**Table 1:** Summary of tide pool temperatures over the 221‐day field incubation, 26 September 2015 through 6 June 2016

Category	Min	Median	Mean	Max
H	5.86	13.08	12.94	22.33
H	5.66	11.43	11.85	23.87
H	4.73	11.33	11.74	22.62
M	6.37	13.08	12.90	20.71
M	6.67	13.08	12.85	20.71
M	6.27	13.46	12.91	20.23
L	6.88	13.17	12.94	21.47
L	8.08	11.33	11.81	19.76
L	7.68	11.14	11.57	19.38

Note

Pools are grouped by categories of high, medium, and low thermal stress.

**Table 2:** Summary of tide pool temperatures in February 2016 and in May 2016

February				
Category	Min	Median	Mean	Max
H	5.86	10.46	10.35	13.94
H	5.66	10.36	10.28	13.76
H	4.73	10.36	10.15	13.65
M	6.37	10.36	10.32	13.08
M	6.67	10.36	10.30	13.56
M	6.27	10.36	10.30	14.42
L	6.88	10.36	10.35	13.65
L	8.38	10.46	10.40	12.98
L	7.98	10.36	10.29	12.40

May				
Size	Min	Median	Mean	Max
S	9.08	11.53	12.09	22.33
S	8.88	11.53	12.06	21.38
S	8.58	11.53	12.30	22.62
M	9.47	11.53	11.92	20.71
M	9.57	11.43	11.74	20.33
M	–	–	–	–
L	9.77	11.43	11.82	20.52
L	10.26	11.43	11.77	19.57
L	10.26	11.33	11.64	19.09

Note

Pools are grouped by categories of high, medium, and low thermal stress.

### 3.2 In situ differences in photosynthetic rate by pool environment

**Figure 3:**
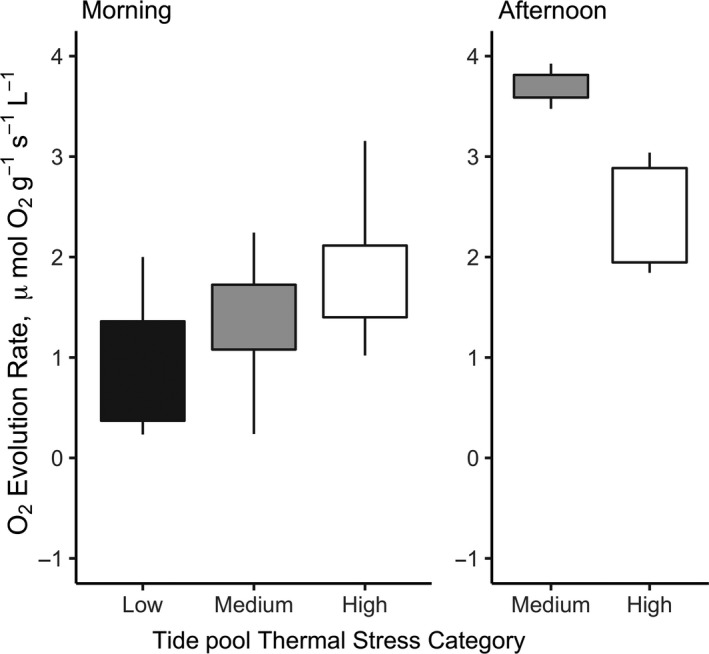
Boxplots of in situ oxygen evolution rate of algal ramets on 6 June 2016. Ramets are grouped by tide pool, labeled categorically as “Low,” “Medium,” and “High” thermal stress

Across pools sampled in both morning and afternoon in the field in June 2016, *E. elongata* exhibited higher photosynthetic rates in the afternoon than in the morning (*F*_1,44_ = 3.24, *p* = .079). Although pools represented different thermal environments, photosynthetic production of *E. elongata* did not differ between pools (Figure 3). There was no effect of thermal stress category on photosynthetic rate within morning samples (*F*_2,32_ = 1.39, *p* = .265).

### 3.3 Effects of treatment temperature and individual identity on photosynthetic rate

**Figure 4:**
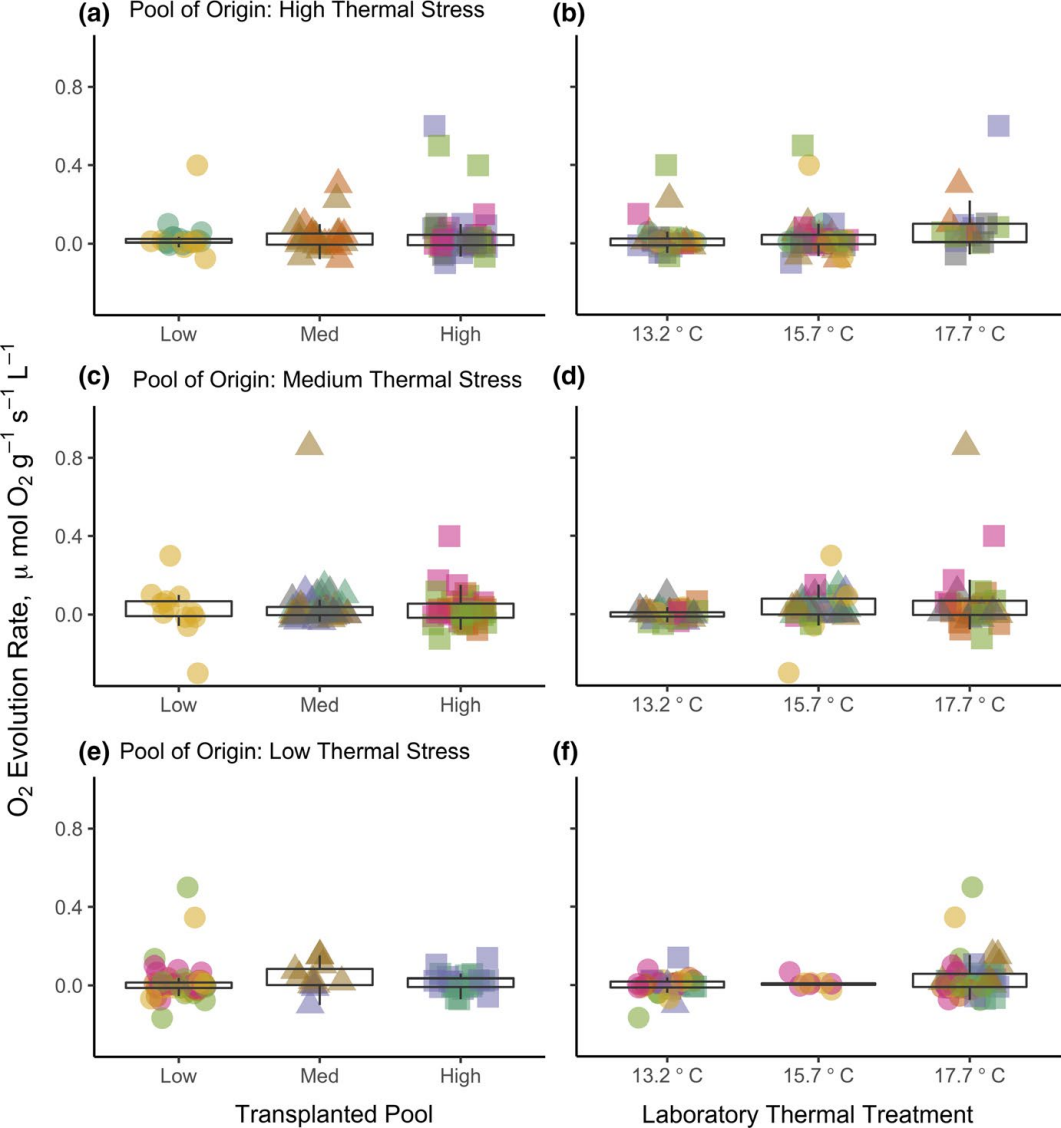
Plots of oxygen evolution rate of algal ramets after 4 weeks in the laboratory incubations. Individual points represent replicates, with individual ramets coded by color and transplanted tide pool coded by plotting character. Panels A and B show data from ramets originally from high thermal stress pools, panels C and D show data from ramets originally from medium thermal stress pools, and panels E and F show data from ramets originally from low thermal stress pools. The first column plots oxygen evolution rate in the laboratory, categorized by transplanted pool (recent environmental history). Tide pools labeled categorically as “Low,” “Med.,” and “High” thermal stress in panels A, C, and E. The second column plots oxygen evolution rate in the laboratory, categorized by laboratory thermal stress treatment (panels B, D, and F). Points are jittered in the x‐direction

Overall, laboratory temperature treatments did not strongly influence photosynthetic rate, and individual identity was not important (Figure 4). We tested for responses to the laboratory treatments over time, with a random effect of individual identity nested within a random effect of tank. We found no effect of sampling date in photosynthetic rate by laboratory treatment, meaning that photosynthetic rate did not increase or decline with time in temperature treatments (all weeks pooled, sampling date as fixed effect; high thermal stress, *F*_3,106_ = 0.664, *p* = .58; medium thermal stress, *F*_3,93_ = 1.38, *p* = .25; low thermal stress, *F*_3,103_ = 1.19, *p* = .32). We also found no effect of individual (all weeks pooled, individual as random effect nested within tank; high thermal stress, *df* = 2,106, Likelihood Ratio Test Statistic (LRT) = 2.98, *p* = .23; medium thermal stress, *df* = 2,93, LRT < 0.001, *p* = 1; low thermal stress, *df* = 2,103, LRT = 0.883, *p* = .64).

We also examined each week separately. After the first week, laboratory treatment temperature did not affect photosynthetic rate (fixed effect, *F*_1,75_ = 2.59, *p* = .31), yet individual identity of each ramet was important (fixed effect, *F*_1,75_ = 2.91, *p* = .092), and no tank effects were detected (random effect, *df* = 2,75, LRT = 2.38, *p* = .32). Treatment temperature did not affect photosynthetic rate in the second week (fixed effect, *F*_1,78_ = 1.25, *p* = .27), individual identity was not important (fixed effect, *F*_1,78_ = 0.81, *p* = .37), and no tank effects were detected (random effect, *df* = 2,78, LRT < 0.001, *p* = 1). In the third week, photosynthetic rate was reduced but variable in the 13.2°C control treatment (fixed effect, *F*_1,77_ = 3.78, *p* = .056), individual identity was not important (fixed effect, *F*_1,77_ = 0.17, *p* = .69), and no tank effects were detected (random effect, *df* = 2,77, LRT < 0.001, *p* = 1). In the fourth week, treatment temperature did not affect photosynthetic rate (fixed effect, *F*_1,77_ = 1.07, *p* = .30), individual identity was not important (fixed effect, *F*_1,77_ = 0.04, *p* = .834; Figure 4), and no tank effects were detected (random effect, *df* = 2,77, LRT < 0.001, *p* = 1).

### 3.4 Effect of environmental history on temperature response

Two timescales of environmental history were tested during this experiment. The pool of origin was representative of long‐term environmental history over the alga's life (including settlement and development), while the pool it was transplanted into accounted for recent environmental history (acclimation over many months). Neither pool of origin nor transplanted pool affected photosynthetic rate in laboratory treatments in week one (fixed effects, pool of origin, *F*_8,71_ = 0.87, *p* = .542, transplanted pool, *F*_8,68_ = 0.66, *p* = .716), week two (fixed effects, pool of origin, *F*_8,71_ = 0.87, *p* = .839, transplanted pool, *F*_8,70_ = 1.24, *p* = .287), week three (fixed effects, pool of origin, *F*_8,68_ = 1.18, *p* = .323, transplanted pool, *F*_8,68_ = 0.59, *p* = .779), or week four (fixed effects, pool of origin, *F*_8,67_ = 0.37, *p* = .931, transplanted pool, *F*_8,68_ = 0.71, *p* = .679; Figure 4).

**Figure 5:**
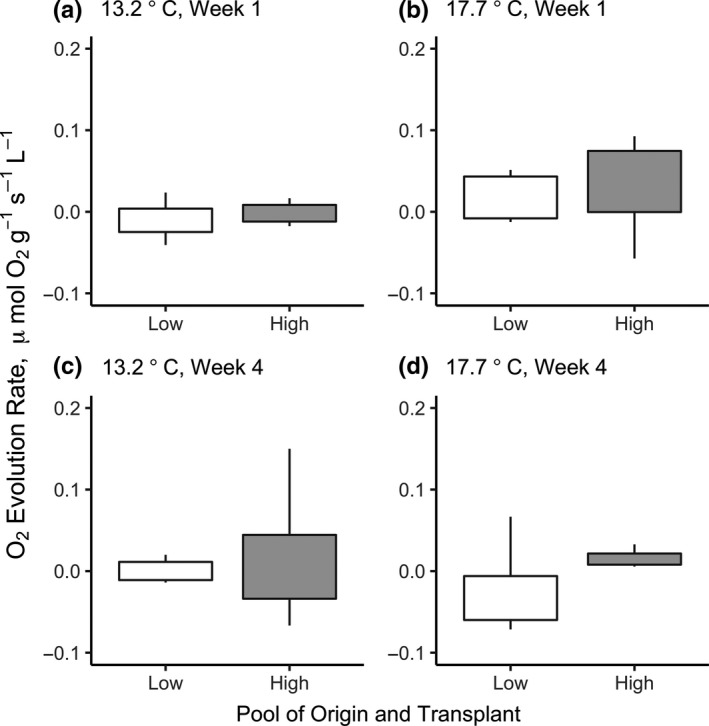
Boxplots of oxygen evolution rate comparing the most extreme environmental history (pool of origin: high vs. low thermal stress) and laboratory treatments (13.2 vs. 17.7°C). This comparison reveals a difference only in once extreme case: higher photosynthetic rate in ramets originally from high thermal stress pools compared to ramets originally from low thermal stress pools, within the 17.7°C during the fourth week of the experiment

To compare the only most extreme cases, we compared photosynthetic rate in ramets originally from high thermal stress pools with rates from ramets originally from low thermal stress pools in the low and high laboratory temperature treatments (13.2 vs. 17.7°C). This comparison revealed a difference only within the 17.7°C treatment in the fourth week of the experiment (ANOVA, *F*_1,8_ = 0.37, *p* = .006; Figure 5).

### 3.5 Within‐individual variation

**Figure 6:**
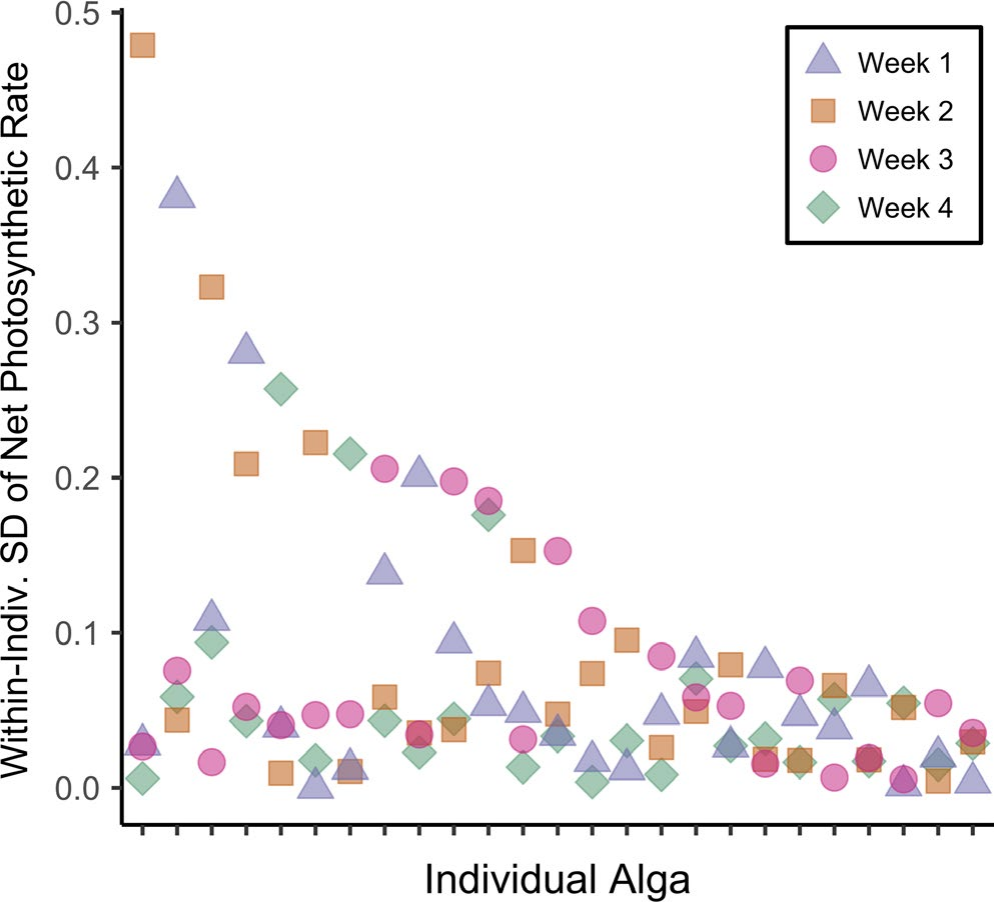
Within‐individual standard deviation (*SD*) of net photosynthetic rate. Individual algae are ordered by maximum *SD* along the x‐axis. *SD* of each individual measured in week 1 shown in blue triangles, week 2 in orange squares, week 3 in pink circles, and week 4 in green diamonds

Within‐individual standard deviation did not change over experimental time, meaning that the effect of individual identity did not become more or less important as individuals acclimatized to their temperature treatments (linear regression, *F*_1,97_ = 0.087, *p* = .77, *r* ^2^ < .001; Figure 6). Standard deviation was not significantly different by individual (ANOVA, *F*_1,97_ = 1.88, *p* = .17). We also quantified within‐individual variation using the range of photosynthetic rates. Like standard deviation, range did not differ over experimental time (linear regression, *F*_1,98_ = 0.073, *p* = .79, *r* ^2^ < .001), nor did it differ by individual (ANOVA, *F*_1,98_ = 3.83, *p* = .053). We note that this analysis is necessarily pooled among temperature treatments because each individual was dispersed between but not within temperature treatment levels.

## 4 DISCUSSION

An individual alga found in a tide pool spends its entire life in the same pool, and thus, the pool's local environment reflects that of the alga's recruitment, development, and lifetime acclimation. While it is likely that dispersal of *E. elongata* occurs between tidal pools, an individual's developmental environment may be particularly important to its future performance under given conditions (Grether, 2005). Given documented physiological differences between subtidal and intertidal species (above), algae inhabiting different pools along a gradient of thermal stress are likely to exhibit different physiological sensitivities. We thus expected the pool of origin to have a lasting effect on algal physiology. We also expected recent environmental history to affect acclimatization to water temperature (Davison, Greene, & Podolak, 1991). The lack of significance in both these factors might be attributable to the temporal patterns of temperature change within tide pools. Although tide pools of different sizes and positions on the shore varied significantly in their exposure to hot and cold temperatures (Figure 2), the duration of extreme temperatures in the intertidal never exceeds a few hours between tides. Within shallow or high pools in the intertidal zone, higher water temperature and irradiance may enhance photosynthesis. Thus, there may be other, nonadaptive mechanisms that minimize stress across intertidal thermal gradients. Additionally, pools that experience the hottest thermal extremes are also those that experience the coldest. Individuals in these pools could thus be acclimatized to greater overall plasticity to thermal stress.

The fact that laboratory treatment temperatures also did not cause any differences in photosynthetic response may suggest that treatments may not have been extreme enough to generate responses. Considering that increases in mean sea surface temperatures are predicted to be driven by increases in short‐term extremes (Alexander et al., 2018), understanding ecophysiological responses to punctuated, anomalous events may be more important than predicting responses to increased, constant temperatures. The goal of this study, however, was not to study stress response, but rather to determine whether increases in near‐future mean temperatures would affect *E. elongata*, intertidal habitat builders, and whether different portions of the environmental mosaic within the intertidal habitat would be differentially affected based on their organisms' environmental history. Such ecophysiological responses may not be tied directly to metabolic rates and photosynthesis, but to population‐ and community‐level changes in competition or ecosystem structure due to range shifts of focal or interacting species (Burrows et al., 2014; Queirós et al., 2015; Vergés et al., 2014). Other studies have found growth sensitivity to nonstressful temperature treatments, though they do not appear tied to differences in photosynthetic activity (Clark, Poore, Ralph, & Doblin, 2013).

Low photosynthetic rates were consistently observed in laboratory conditions relative to field‐measured rates. When stressed, metabolic can exceed oxygen produced by photosynthesis. However, as discussed above, lack of differential response between laboratory temperature treatments indicates that this may not have been the case in our experiments. Another likely explanation is reduced irradiance between field and laboratory conditions, which may have limited oxygen production in the laboratory and potential limited the potential for individuals to reach full photosynthetic capacity at optimal temperatures (e.g., Egilsdottir et al., 2015). The photosynthetic patterns observed in field measurements (Figure 3) bolster this theory, revealing elevated photosynthetic output in the afternoon while irradiance is an order of magnitude greater than in the morning. While our PAR levels in the laboratory matched in situ mean summer subtidal levels in the Southern UK (Kolzenburg et al., 2019), mean summer PAR may be chronically undersaturating in this region, and our field measurements may represent an anomalously productive day, as we biased these measurements for sunny conditions that allowed use of electronics in the intertidal zone.

Variability between individuals is emerging as an important theme in climate change studies due to its role as a driver of resilience or adaptation within populations and species (Calosi et al., 2017; CaraDonna, Iler, & Inouye, 2014; Inouye, 2008; Kroeker, Kordas, Crim, & Singh, 2010; Ovaskainen et al., 2013; Vargas et al., 2017). However, not only does individual variability exist in climate change responses, it may affect ecological function under climate stress (CaraDonna et al., 2014; McCoy & Kamenos, 2018; McCoy, Kamenos, Chung, Wootton, & Pfister, 2018). Trait variability, as a trait in itself, can have a genetic basis (Clark et al., 2013; Pistevos, Calosi, Widdicombe, & Bishop, 2011), affecting species specialization and adaptation potential to new or changing environments (Kawecki, 2008; Wiens & Graham, 2005). Such variation in temperature response could influence spatial patterns of temperature susceptibility if populations are not genetically well mixed, or between populations, though apparently not on the pool‐by‐pool scale of the experiments conducted in this study. As revealed in our dataset, high variability in climate change responses between individuals may indicate the potential for resilience to future conditions and, thus, may play a compensatory role at the population or species level. Within other intertidal macroalgal species, individuals belonging to the same genotype perform consistently, and those that are more productive at elevated temperatures are also more productive in control temperatures (Clark et al., 2013).

In contrast, we found that replicate ramets, presumably belonging to the same individual, were not consistent in their photosynthetic performance between laboratory treatments. We found evidence for variability among individuals. Some individuals performed consistently between experimental treatments during the first week of the laboratory temperature treatment. This may indicate that short‐term acclimatization to laboratory conditions was individual‐dependent, although it did not differ by recent or long‐term thermal history, as we had expected. Interestingly, this also did not necessarily translate to consistency in the amount of within‐individual variability, meaning that replicates of the same individual were more or less similar in their photosynthetic performance from one experimental time point to another, with no clear trend in variability over experimental time (Figure 3).

In this study, we found strong evidence that *E. elongata* individuals were plastic in their photosynthetic response to thermal stress. This claim is based on the similar responses of individuals from different long‐term thermal environments, including settlement and development (represented by pool of origin), different recent environments (acclimation over 7.5 months in transplanted pools), and when exposed to different temperature treatments in the laboratory. Based on the regular variation in thermal stress in the intertidal zone, it is possible that pool‐dwelling *E. elongata* are simply acclimated to thermal variability, rather than to a specific degree of variability. This argument is consistent with other aspects of macroalgal ecology. For example, carbon concentrating mechanisms are constantly upregulated in thermally variable habitats (Stepien, 2015), including the intertidal zone (Murru & Sandgren, 2004; Raven & Osmond, 1992). The fact little difference was observed over the duration of our laboratory experiment also points to the high plasticity of intertidal *E. elongata* photosynthetic response to a range of thermal conditions—including adjustment from highly variable field conditions to the stable laboratory environment. Species‐specific resiliency to climate changes will drive the reassembly of changing communities, and therefore, resilience of habitat‐forming species like *E. elongata* may facilitate transitions between functional community states. Evaluating the relative contributions of genetics and different temporal scales on which acclimation and plasticity act within intertidal organisms may provide insights into the process of the evolution of plasticity and its importance in determining population‐scale responses to ongoing climate changes.

## CONFLICT OF INTEREST

The authors declare no competing interests.

## AUTHOR CONTRIBUTIONS

SJM designed and conducted the experiment, analyzed the data with guidance from SW. SJM wrote the manuscript with input from SW. Both authors obtained funding for this research.

### OPEN DATA BADGES

This article has earned an https://openscience.com for making publicly available the digitally‐shareable data necessary to reproduce the reported results. The data is available at https://doi.org/10.5061/dryad.ht76hdr9x.

## Data Availability

Environmental and experimental datasets are available for download at Dryad, https://doi.org/10.5061/dryad.ht76hdr9x.
